# The Transcriptional Response of Soil Bacteria to Long-Term Warming and Short-Term Seasonal Fluctuations in a Terrestrial Forest

**DOI:** 10.3389/fmicb.2021.666558

**Published:** 2021-08-27

**Authors:** Priyanka Roy Chowdhury, Stefan M. Golas, Lauren V. Alteio, Joshua T. E. Stevens, Andrew F. Billings, Jeffrey L. Blanchard, Jerry M. Melillo, Kristen M. DeAngelis

**Affiliations:** ^1^Department of Biology, Keene State College, Keene, NH, United States; ^2^Department of Microbiology, University of Massachusetts Amherst, Amherst, MA, United States; ^3^Organismic and Evolutionary Biology, University of Massachusetts Amherst, Amherst, MA, United States; ^4^Department of Biological Sciences, University of South Carolina, Columbia, SC, United States; ^5^The Ecosystems Center, Marine Biological Laboratories, Woods Hole, MA, United States

**Keywords:** meta-transcriptomes, microbial, terrestrial, carbon cycle, global warming

## Abstract

Terrestrial ecosystems are an important carbon store, and this carbon is vulnerable to microbial degradation with climate warming. After 30 years of experimental warming, carbon stocks in a temperate mixed deciduous forest were observed to be reduced by 30% in the heated plots relative to the controls. In addition, soil respiration was seasonal, as was the warming treatment effect. We therefore hypothesized that long-term warming will have higher expressions of genes related to carbohydrate and lipid metabolism due to increased utilization of recalcitrant carbon pools compared to controls. Because of the seasonal effect of soil respiration and the warming treatment, we further hypothesized that these patterns will be seasonal. We used RNA sequencing to show how the microbial community responds to long-term warming (~30 years) in Harvard Forest, MA. Total RNA was extracted from mineral and organic soil types from two treatment plots (+5°C heated and ambient control), at two time points (June and October) and sequenced using Illumina NextSeq technology. Treatment had a larger effect size on KEGG annotated transcripts than on CAZymes, while soil types more strongly affected CAZymes than KEGG annotated transcripts, though effect sizes overall were small. Although, warming showed a small effect on overall CAZymes expression, several carbohydrate-associated enzymes showed increased expression in heated soils (~68% of all differentially expressed transcripts). Further, exploratory analysis using an unconstrained method showed increased abundances of enzymes related to polysaccharide and lipid metabolism and decomposition in heated soils. Compared to long-term warming, we detected a relatively small effect of seasonal variation on community gene expression. Together, these results indicate that the higher carbohydrate degrading potential of bacteria in heated plots can possibly accelerate a self-reinforcing carbon cycle-temperature feedback in a warming climate.

## Introduction

Terrestrial ecosystems play a vital role in the global carbon (C) cycle as soils are estimated to store twice as much C as the atmosphere and all vegetation combined (Schimel, 1995; Scharlemann et al., 2014). Soil C-dynamics is however tightly coupled to temperature changes making C sinks vulnerable to global warming. Studies have shown that warming-induced increases in soil respiration have the potential to convert soil from a C-sink to a C-source triggering a self-reinforcing C cycle-temperature feedback (Friedlingstein et al., 2006; Allison and Treseder, 2011; Tucker et al., 2013). Microbes play a pivotal role in soil C processes, acting to process incoming C to form stable soil organic matter (SOM) pools (Baldrian et al., 2012; Lladó et al., 2017) as well as acting to destabilize stored soil organic carbon (Bradford et al., 2008; Feng et al., 2008; Pisani et al., 2015). Increasing temperature as a result of climate change has significantly affected the microbial communities and microbial functions (Castro et al., 2010; Gray et al., 2011; Sheik et al., 2011; Zhou et al., 2012). However, we do not yet understand the microbial mechanisms driving warming-induced soil C losses well enough to predict these dynamics in a warming climate.

One challenge in identifying the mechanisms associated with microbial-driven soil C loss is the inherent variability in short- vs. long-term responses to warming. There is growing evidence that with long-term warming, there is a gradual decrease in warming effects on soil respiration (Luo et al., 2001, Melillo et al., 2002, 2017; Kirschbaum, 2004; Romero-Olivares et al., 2017). For example, in an ongoing soil warming experiment at the Harvard Forest in central Massachusetts, soils that are warmed 5°C above ambient temperatures fluctuated from being initially higher (Phase I; 1–10 years), then invariant (Phase II; 11–17 years), then higher (Phase III; 18–23 years) and currently approximately equal to (Phase IV) in respiration compared to control plots (Melillo et al., 2002, 2017). Such time-dependent variations can be due to acclimation of soil microorganisms to the increased temperature changes (Bradford et al., 2019; Dacal et al., 2019) and/or functional shifts in microbial community associated with decomposition of labile and recalcitrant C-pools based on their relative availability (Hartley et al., 2009; Schindlbacher et al., 2015; Pold et al., 2016).

Our recent work in Harvard Forest suggests that chronic warming has led bacteria to play an increasing role in SOM turnover (Frey et al., 2008; DeAngelis et al., 2015). Around 26 years of simulated warming was associated with sustained reduction in fungal biomass but not bacterial biomass (Frey et al., 2008; DeAngelis et al., 2015). We observed warming-accelerated respiration despite declining quality and quantity of SOM (Bradford et al., 2008; Pold et al., 2015, 2017). Periods of soil carbon decay were punctuated by periods of change in the microbial communities (Melillo et al., 2017), though the bacterial community composition did not appear to have changed substantially. Because two-thirds of soil respiration is induced by microbial activities (Melillo et al., 2002, 2011), we predicted that changes in microbial gene expression, as measured by meta-transcriptomics, could potentially detect the microbial mechanism of warming accelerated soil C loss observed in these forests. Further, studies have shown that short-term warming induces stress responses in bacteria triggering metabolic adjustments in protein productions (García-Descalzo et al., 2014) and growth vs. maintenance trade-offs by modifying their carbon use efficiency (CUE; Rodríguez-Verdugo et al., 2014, Pold et al., 2020). Taken together, these studies indicate the importance of studying the functional shifts in microbial communities experiencing long-term chronic warming. Inquiries as such can provide important insights on precise functional processes that dominate the energetic budgets of accelerated soil C loss in the face of climate change.

Along with long-term warming, annual temperature fluctuations due to seasonal variations in temperate forests can significantly alter the soil microbial community composition (Habekost et al., 2008; Cao et al., 2011) and the C allocation to soil microbes (Žifčáková et al., 2017). Few studies have looked into how seasonal variation in C allocation and microbial community composition can impact soil C dynamics (Lladó et al., 2017). Chronic soil warming was shown to have seasonal effects on soil moisture, respiration, and net nitrogen (N) mineralization (Contosta et al., 2011), suggesting that there is likely a seasonal component to chronic warming-accelerated soil carbohydrate degradation. Based on microarray analysis of soils from a grassland warming experiment, warming-associated gene expression was altered globally in warming treatments, with C degradation genes altered significantly with soil moisture (Xue et al., 2016). However, the relative importance of seasonality and its ultimate impact on microbially-accelerated soil C-dynamics in temperate forests is still largely unknown.

In this study, we applied techniques in meta-transcriptomics to identify changes in bacterial structure and function when exposed to both seasonal fluctuations and long-term simulated warming in Harvard Forest, MA. We first compared the magnitude of seasonal vs. long-term effects of warming on the active bacterial communities to understand long-term changes in C-energetics in this forest. Second, we identified metabolic pathways that showed a prominent “warming effect” to understand processes that facilitated the observed warming responses in these communities over time. Specifically, we tested the hypothesis that accelerated mineralization of recalcitrant C pools is due to a shift in the microbial community structure and functional potential. Finally, we used quantitative gene expression data to generate new hypotheses about the mechanistic nature of microbial adaptation of warming that extends beyond the expected increased utilization of soil carbon pools. Meta-transcriptomic data is limited to providing a relative account of the active transcripts, which can lead the libraries to be dominated by metabolic pathways shared by most cells instead of representing unique expression pathways characteristic of the given environmental conditions (Gifford et al., 2011). To overcome this, we employed the use of an internal standard to estimate the absolute number of transcripts in our samples compared to the number of transcripts sequenced (Gifford et al., 2011; Satinsky et al., 2013). Because of the previously observed changes in soil bacterial and fungal abundances, absolute measures of transcripts can help elucidate how gene expression might be linked to chronic warming.

## Materials and Methods

### Study Site and Sample Collection

Soils for this study were collected from the Prospect Hill warming site at the Harvard Forest Long Term Ecological Research Site in Petersham, Massachusetts, United States (42.54°N, 72.18°W). The Prospect Hill experimental site has been studied as part of a long-term warming experiment in this forest for over 25 years. The soils at this site have been heated 5°C above ambient soil temperatures since 1991 using buried resistance cables to 10 cm depth (Peterjohn et al., 1994). The experimental design consists of six replicated 6 m by 6 m plots in a randomized block design. Each block contains a heated plot, a control plot with cables that are not heated and an additional control plot with no underground cables. The soil has a distinguishable upper organic horizon layer and a deeper mineral layer. Extensive information regarding geographic location, soil conditions, and physical attributes, carbon dioxide fluxes, microbial community structures have been previously published (Melillo et al., 2011; DeAngelis et al., 2015; Pold et al., 2017). To study the effect of long-term warming and short-term seasonal fluctuations on soil microbial communities, we collected a total of 32 soil samples from the Prospect Hill study site that were collected from two treatment plots [heated and control (with cables turned off)], two soil types (organic and mineral) and two time points [3rd June 2014 (T2) and 28th October 2014 (T6)]. At T2, the average temperature of the heated and control plots was 20.37 and 15.87°C and at T6, it was 17.53 and 11.97°C, respectively. Each site or treatment had four soil replicates. Sample cores (9–10 cm deep) were collected using one-half inch diameter stainless steel corer (top ~1 cm: organic soil; bottom 1–10 cm: mineral soil), sterilized with 70% ethanol and alternating between heated and control plots to reduce collection biases. Soil cores were separated into mineral and organic horizons, followed by homogenization by hand, after which soil samples were separately flash frozen using a dry ice ethanol bath within about 10 min of collection. Frozen soil samples were transported back to the lab, where they were kept at −80°C until RNA extraction.

### RNA Extractions and Purifications

For all 32 samples, total RNA was extracted from about ~2 g of soil using Mo-Bio RNA PowerSoil Total RNA isolation kit (MoBio, Carlsbad CA, United States) following the manufacturer’s protocol with slight modifications. Soil samples were added to the tubes after Bead Solution, SR1, SR2, and phenol:chloroform:isoamyl alcohol to reduce contamination with RNases. The RNA was incubated at 65°C for 5 min, while vortexing every minute to allow for better solubilization of RNA. Samples were frequently pushed through the capture columns using a syringe barrel, ensuring the flow rate did not exceed one drop per second, as suggested in the troubleshooting guide. DNA was removed from extracted RNA using the MoBio DNase Max Kit (formerly the RTS DNase kit; MoBio, Carlsbad CA, United States) according to instructions. RNA was quantified using the Qubit RNA BR assay kit (Thermo Fischer Scientific Inc., Waltham, MA, United States) in a Qubit 2.0 Fluorometer (Invitrogen, Waltham MA, United States) before being stored at −80°C. Prior to RNA extraction, we added a known amount of internal RNA standard (~0.5% of estimated RNA yield; Satinsky et al., 2013) to each of our samples to calculate the absolute number of transcripts present per unit mass of soil (i.e., count gm^−1^). The RNA standard was synthesized *in vitro* using methods described in Appendix 1 following protocols in Gifford et al. (2011).

### cDNA Library and Quantification

Purified RNA (10–100 ng) from above was used in construction of cDNA library using NEBNext® Ultra™ Directional RNA Library Prep Kit for Illumina (New England BioLabs, Ipswich MA, United States) for all 32 samples following manufacturer’s protocol with fragmentation time of 8 min and 12 PCR cycles. Each library was generated using a unique 8 bp multiplex barcode provided in an accessory kit (NEBNext Multiplex Oligos for Illumina). Following library construction, each sample was quantified using the Quant-iT PicoGreen double-stranded DNA (dsDNA) assay kit (Invitrogen, Waltham MA, United States), according to the product instructions. Library size distribution was determined using a BioAnayzer 2100 (Agilent Technologies, Santa Clara CA, United States) with DNA HighSensitivity chips and reagents (Agilent Technologies, Santa Clara CA, United States). The region average from the bioanalyzer results were then used to quantify the concentrations of individual libraries with the help of a qPCR based assay using NEBNext® Library Quant Kit for Illumina (New England BioLabs, Ipswich MA, United States). Following quantification, all libraries were diluted to 4 nM using 0.1 TE buffer and four of these libraries were pooled together in equimolar concentrations to run as a single sample in the Illumina NextSeq 500 sequencing platform (Illumina, San Diego CA, United States). A total of eight runs were done for all 32 samples. For sequencing, we used Illumina NextSeq 500/550 High Output v2 kit (300 cycles) that generated paired-end 150 bp reads (Illumina, San Diego CA, United States).

### Sequence Data Processing and Annotation

Base scores and adapter trimming were performed in BaseSpace (Illumina, San Diego CA, United States), where individual samples were binned based on the multiplex barcode prefix. Initial quality checks of sequences were performed in *FastQC* (Andrews, 2010) following trimming of low quality sequences (Q < 33) using *Trimmomatic* (v 0.27; Bolger et al., 2014). Illumina specific adapter sequences were also removed. Following quality check, paired end reads were merged using FLASH 2.0 (Fast Length Adjustments of Short Reads) with default settings except fragment length was set to 150 bp (Magoč and Salzberg, 2011). rRNA sequences were identified and removed from the merged reads using SortmeRNA (Kopylova et al., 2012) using their eight prepackaged rRNA databases [SILVA SSU Ref NR v. 119 (bac 16S; arc 16S; euk 18S); SILVA LSU Ref v. 119 (bac 23S; arc 23S; euk 28S); RFAM (5S; 5.8S)]. Remaining non-rRNA sequences were then blasted against the NCBI non-redundant protein database to identify putative mRNA sequences using DIAMOND (Buchfink et al., 2015). Matches with *e*-value less than or equal to 1e^−5^ were retained for further analyses. Putative mRNAs were then taxonomically and functionally annotated in MEGAN (parameters: minimum bit score, 50; minimum support, 1; top percent 10; Huson et al., 2007). Functional annotation of mRNAs was done using the KEGG classification systems in MEGAN.

### Carbohydrate-Active Enzyme Annotation

To understand the effect of warming on microbial decomposition and metabolism, we used putative mRNA transcripts from above to annotate them based on the carbohydrate-active enzyme (CAZy) database that includes enzymes that degrade, modify and create glycosidic bonds. Putative mRNA reads were first translated into six reading frames using *Transeq* program within EMBOSS 6.4.0 package (Rice et al., 2000). The resulting amino acid sequences were then queried against a CAZy database (Lombard et al., 2014) obtained from the dbCAN program (Yin et al., 2012) using HMMER (Finn et al., 2011; v. 3.1b2). Only matches with *e*-value less than or equal to 1e^−5^ were used in further analyses.

### Taxonomic Composition of the RNA Transcripts

To identify changes in bacterial community composition in response to long-term warming and short-term seasonal fluctuations, we determined the relative abundance of taxa in all samples in two different ways. First, the putative rRNA sequences identified by SortmeRNA (Kopylova et al., 2012) from above were aligned to the default Silva 128 SSU Ref Nr99 reference database using the phylogeny assignment program MATAM (Pericard et al., 2018). This program uses an RDP classifier (Wang et al., 2007) for assignment of taxa. Second, the putative mRNA reads identified by DIAMOND (Buchfink et al., 2015) from above were taxonomically annotated in MEGAN software package (Huson et al., 2007) using the least common ancestor (LCA) algorithm.

### Statistical Data Analyses

All analyses were performed in the statistical platform R (v 3.6.2; R Core Team, 2013). One of the samples had poor sequencing success (<1,000 sequences) and was removed from further analyses. To account for differences in sampling depths, each sample. KEGG and CAZy annotated transcripts and MATAM assigned phylum abundances were visualized by non-metric multidimensional scaling (nMDS) ordination using Bray-Curtis dissimilarity matrix using the *vegan* library in R (Dixon, 2003; 1,000 iteration for a two-dimensional solution – final stress KEGG: 0.123; CAZy: 0.0856; Phylum: 0.20). Sample clustering was analyzed for significance using a three-factor multivariate PERMANOVA with 1,000 permutations following square-root transformation of all transcripts. The model had three fixed factors and their interactions – treatment (heated and control); horizon (organic and mineral), and time-points (T2 and T6). Cohen’s d estimation was used to test the effect of treatment (H vs. C), horizon (O vs. M), and time points (T2 vs. T6) on transcript and phylum abundances. To account for differences across samples, all abundances were normalized to the number of reads per million annotated mRNA in each sample. Three-way ANOVA was used to test the treatment, horizon, and time-points on RNA yields and annotated transcripts. Assumptions of normality were validated and Levene’s Test for equality of variance were tested with no significant variation observed. Differential expression of KEGG annotated metabolic genes between heated and control plots were calculated using *edgeR* in R (Robinson et al., 2010). In order to further elucidate the association between differential expressions of metabolic genes and observed metabolic enzymes activities in the soil microbiome, weighted gene correlation network analyses were done in R (Langfelder and Horvath, 2008). The enzyme activity data was obtained from Pold et al. (2017), as these samples were collected at the same time and at the same study site. Additionally, we used total oxygen, water content, dry and wet weight of the sediments reported in the same study to identify any correlation between gene expressions and physical properties of the soil. Metabolic gene list was obtained from transcripts annotated under the “metabolism” category in KEGG. Minimum number of genes in each module was set to 25 and a 0.7 threshold was used to merge similar modules.

## Results

### Sequence Quality and Data Set Description

An average of 111 M high quality sequence reads per sample were obtained with a range from 14 to 200 M reads for individual samples (Table 1). Since, we did not use rRNA depletion during our library construction, rRNA reads made up an average of 91.03% of our total sequences (71.7–95%). Putative mRNA sequences identified from the NCBI non-redundant protein database accounted for 1.18% or 1.5 M sequences across samples (0.01–7.38%; 0.1–1.2 M). One sample (T2, Control, and Organic) showed an unusually high percentage (81.68%, 12 M) of putative mRNA reads for a non rRNA depleted sample. Hence, it was omitted from all subsequent analyses. We found no significant differences in the yield of putative mRNA sequences between our treatments (heated vs. control), time points (T2 vs. T6), and soil types (Organic vs. Mineral; See Supplementary Data: Appendix 2A).

**Table 1:** Sequencing output – RNA sequencing results with percentages of rRNA, mRNA, and number of internal standard reads recovered from each sample.

Site	Time points	Treatments	Soil types	Replicates	Total reads	rRNA (%)	mRNA reads	mRNA (%)	Internal standard reads
Prospect Hill	T2 (3rd June)	Heated	Organic	1	122,445,353	92.37	776,829	0.63	40,167 (0.033)
				2	119,068,820	95.49	874,119	0.73	27,342 (0.023)
				3	121,681,637	91.97	1,062,698	0.87	37,597 (0.031)
				4	110,749,650	93.5	1,004,683	0.91	27,443 (0.025)
			Mineral	1	200,636,302	93.87	1,600,486	0.80	37,076 (0.0001)
				2	106,671,365	86.34	751,973	0.70	148 (0.000)
				3	150,911,448	93.17	1,429,743	0.95	11,675 (0.078)
				4	14,366,071	95.32	100,969	0.70	799 (0.006)
		Control	Organic	1	174,072,328	95.01	1,266,671	0.73	21,913 (0.013)
				2	84,722,020	87.73	2,720,341	3.21	36,926 (0.043)
				3	96,103,877	93.01	834,364	0.87	37,048 (0.039)
				4	103,877,062	92.61	819,071	0.79	3,577 (0.003)
			Mineral	1	118,169,425	78.73	1,650,805	1.40	135 (0.000)
				2	126,980,438	92.86	1,026,399	0.81	32,620 (0.026)
				3	117,483,686	88.15	1,608,598	1.37	341 (0.000)
				4	51,771,336	18.32	12,438,133	24.03	1,661 (0.003)
	T6 (28th October)	Heated	Organic	1	88,248,858	88.54	1,013,469	1.15	1804 (0.002)
				2	74,353,265	92.62	508,318	0.68	13,097 (0.018)
				3	115,840,435	85.7	1,206,587	0.01	12,966 (0.011)
				4	113,836,625	93.63	1,028,287	0.90	30,889 (0.027)
			Mineral	1	101,565,281	71.70	1,523,539	1.50	79 (0.00)
				2	49,534,972	93.07	290,162	0.59	5,536 (0.011)
				3	121,535,724	93.15	1,405,430	1.16	3,405 (0.003)
				4	102,602,888	93.93	775,689	0.76	1911 (0.001)
		Control	Organic	1	193,899,095	93.39	1,673,111	0.86	25,323 (0.013)
				2	131,247,689	92.39	2,352,189	1.79	63,261 (0.048)
				3	49,240,403	93.07	297,382	0.60	7,448 (0.015)
				4	119,163,187	94.39	967,338	0.81	32,103 (0.027)
			Mineral	1	93,916,386	82.69	1,498,979	1.60	75 (0.00)
				2	153,163,875	94.59	1,130,284	7.38	30,679 (0.020)
				3	142,257,923	95.44	877,431	0.62	11,868 (0.008)
				4	96,556,916	93.40	816,565	0.85	8,982 (0.009)

In the internal standard read column, values within () indicates % of internal standards sequence to the total number of putative mRNA transcripts.

The internal standard copy number averaged at 15,000 across samples (75–63,000), which is within the expected range (0.01–0.04%; Satinsky et al., 2013; Table 1). Based on the recovered internal standard sequences, the sequence depth was estimated to vary from 2 × 10^−27^–8 × 10^−27^, which is significantly lower than the previously reported values from aquatic systems (~1.5 × 10^−10^; Gifford et al., 2011). To our knowledge, this is the first study that estimated sequence depth with the use of internal standards in a soil microbial study system. No significant differences were found in the number of internal standard sequences between our treatments (heated vs. control), time points (T2 vs. T6), and soil type (Organic vs. Mineral; See Supplementary Data: Appendix 2B). However, since the number of internal sequences in four samples were very small (75–148; Table 1), we did not include total transcript measurements in subsequent analyses.

### Global Gene Expression

An average of 1.16 M putative mRNA sequence reads was obtained per sample (~0.15% of the total reads; Table 1), which included reads that had a significant match to the NCBI non-redundant protein database (Table 1). Across all samples, 58.40% of the putative mRNA sequences were functionally annotated to the KEGG database and about 5.24% of the reads could be attributed to CAZy classes (~85,000 reads per sample). Similar to previous analyses, we found no significant difference in the proportion of annotated reads between our treatments (heated vs. control), time points (T2 vs. T6), and soil type (Organic vs. Mineral; See Supplementary Data: Appendices 2C,D). We mapped rarefied read abundances to metabolic pathways in the KEGG database in MEGAN to explore the metabolic range of transcripts in our study. We found nearly complete coverage for major metabolic pathways, suggesting sufficient sequencing depths in our samples (See Supplementary Data: Appendix 3).

### KEGG Annotated Transcripts

**Figure 1:**
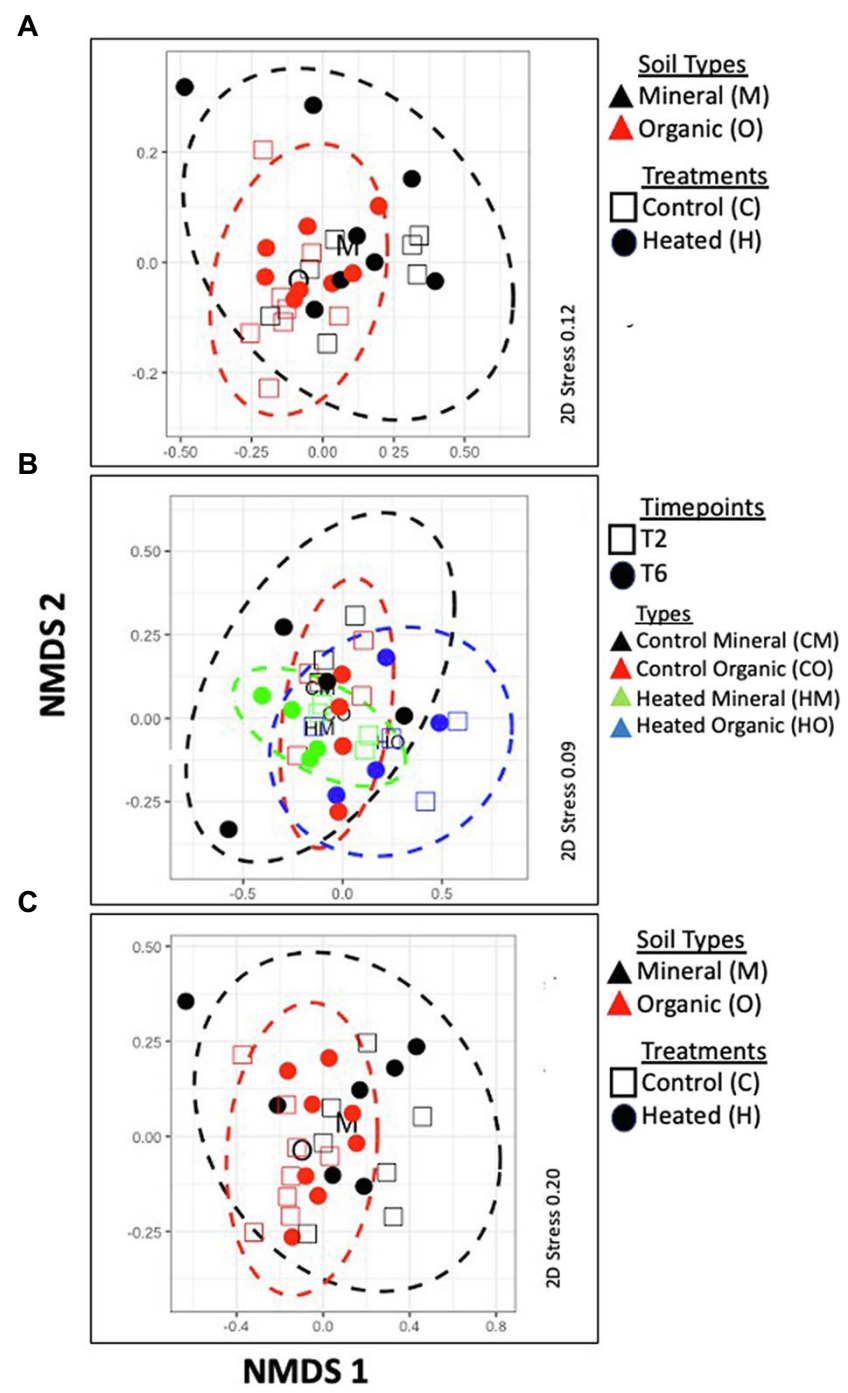
Non-metric multidimensional scaling (nMDS) of the RNA-Seq data – **(A)** based on KEGG transcripts; **(B)** CAZy transcripts and **(C)** Phyla abundances. KEGG and Phyla abundances are clustered by soil types [organic (O) vs. mineral (M)] and treatments [Heated (H) vs. Control (C); KEGG and Phyla] and CAZy transcripts are clustered by soil types, treatments, and time points (T2 and T6). Correlations with grouped samples were calculated by a three-factor multivariate PERMANOVA analyses based on Bray-Curtis similarity that revealed significant differences between soil types (organic vs. mineral) but no differences between time points (T2 vs. T6) or treatments (heated vs. control) in all three groups.

**Figure 2:**
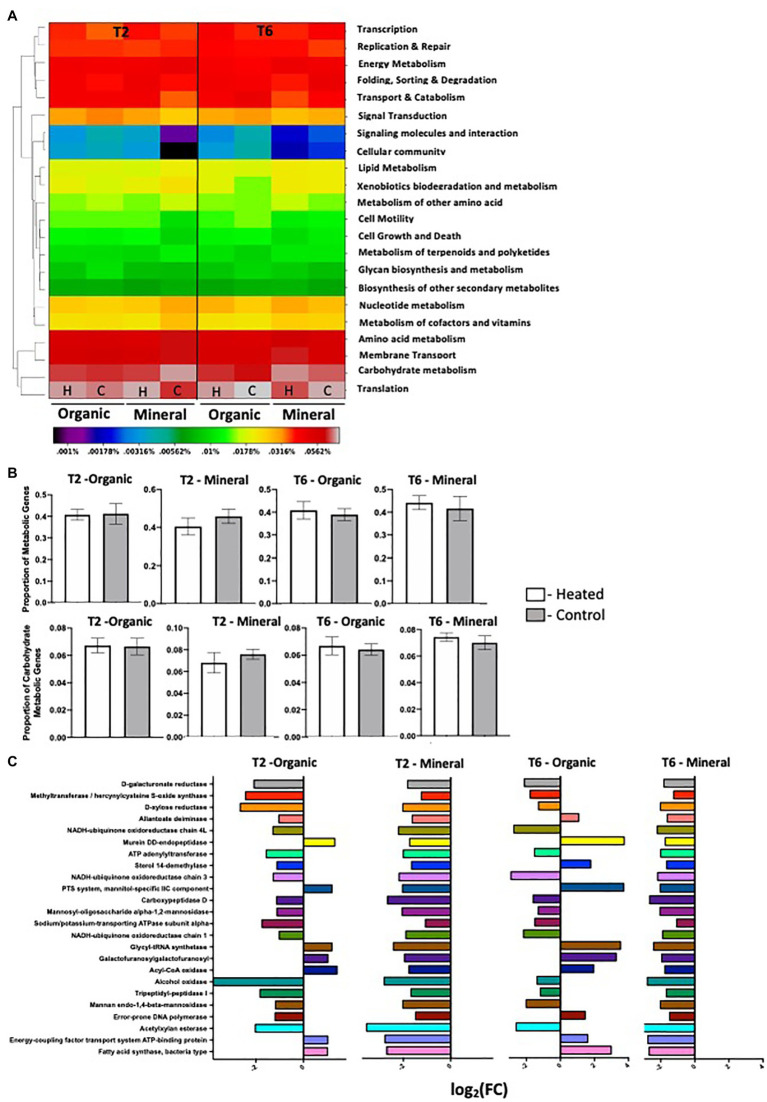
KEGG annotations – **(A)** heat map showing the mean relative abundances of major KEGG categories in annotated transcripts across all eight treatment groups (H-Heated; C-Control). The dendrogram clusters the categories by common expression patterns using a two-way hierarchical clustering using the complete linkage method; **(B)** Mean relative abundances of transcripts annotated in KEGG metabolism category (Top Panel) and KEGG carbohydrate metabolism division (Bottom Panel); **(C)** Genes that are differentially expressed between heated and control plots in organic and mineral horizons in T2 and T6 time points. *Edge R* analyses was done on KEGG transcripts that were annotated as genes involved in metabolism. Differential expression is measured as log of the fold change ratio (FC). So, a log_2_FC of +2 means twice the abundance of the gene in heated plots compared to control, while a log_2_FC of −2 indicates twice the abundance of the gene in control plots compared to heated. All differential expression is measured at FDR 5%. Note that +ve and −ve FC ratios are relative to the specific heated and control plots that are used in the pair-wise comparisons.

Overall, analysis of global gene expression as viewed by KEGG pathway annotation suggests a small but detectable warming treatment effect. Treatment (H vs. C) had the highest effect size as indicated by Cohen’s d effect size estimation, followed by horizon and season (Table 2). PERMANOVA analyses showed a significant effect of horizon (M vs. O) with no interaction between treatment, horizon, and time-point (Table 3). nMDS ordination showed no overall partitioning of the samples by treatment (H vs. C) or by time-points (T2 vs. T6; Figure 1A). Abundances of transcripts belonging to major KEGG categories (Figure 2A) and “Metabolism” and “Carbohydrate metabolism” divisions (Figure 2B) showed no observable variation between heated vs. control plots. However, when we mapped abundances of individual transcripts annotated within the “Metabolism” division in iPATH (Letunic et al., 2008), we detected a positive warming effect in major pathways (increased abundances in heated plots) involved in fatty acid metabolism, glutathione metabolism, and nucleotide metabolism (See Supplementary Data: Appendix 4). Therefore, we conducted *edge R* analyses to look at pair-wise comparison of differential gene expression between heated and control plots (Figure 2C). Most differentially expressed genes in these pairwise comparisons were unique, and genes commonly expressed across samples made up only 0.6–2.4% of the annotated transcripts (See Supplementary Data: Appendix 5), which was consistent with our ordination analyses (See Supplementary Data: Appendix 4A). Most metabolic genes annotated by KEGG pathways showed a lower relative abundance in the heated plots compared to the controls (Figure 2C). Warming-induced enrichment of transcripts were only observed in the organic horizon. Murein DD-endopeptidase, mannitol-specific ICC component, tRNA synthetase, Galactofuranosyl synthetase, Acetyl CoA, and Fatty Acid Synthase are few enzymes that showed increased abundances in the heated plots. Most of these enzymes are related either to carbohydrate or fatty acid metabolism pathways indicating differences in microbial C dynamics between heated and control plots. Relatively few transcripts that were abundant in control plots were related to lipid and/or carbohydrate metabolism (Figure 2C).

**Table 2:** Effect size calculation – effect size estimation (*η*^2^) on transcripts annotated by KEGG and carbohydrate-active enzyme (CAZy) databases and phylum abundances annotated by MATAM.

Factors	KEGG (Metabolism)	CAZy (All)	OTUs
Treatment (Heated vs. Control)	4.26	4.28	3.67
Soil types (Organic vs. Mineral)	2.80	10.30	11.49
Time points (T2 vs. T6)	1.30	1.70	1.41

**Table 3:** Multivariate analyses – three-factor multivariate PERMANOVA analyses based on Bray-Curtis similarity of KEGG transcripts, CAZy transcripts, and phylum abundances.

Source	df	MS	Value of *p*
KEGG			
Treatment (Heated vs. Control)	1	156.82	0.392
Soil types (Organic vs. Mineral)	1	472.85	0.002^*^
Time points (T2 vs. T6)	1	104.71	0.929
Treatment × Soil types	1	136.18	0.561
Treatment × Time points	1	202.58	1.2902
Soil types × Time points	1	115.05	0.797
Treatment × Soil types × Time points	1	116.15	0.779
Residual	23	157.01	
Total	30		
CAZy			
Treatment (Heated vs. Control)	1	692.9	0.061
Soil types (Organic vs. Mineral)	1	1649.3	0.004^*^
Time points (T2 vs. T6)	1	184.06	0.791
Treatment × Soil types	1	456.89	0.183
Treatment × Time points	1	355.39	0.294
Soil types × Time points	1	475.75	0.152
Treatment × Soil types × Times points	1	180.41	0.809
Residual	23	312.83	
Total	30		
Phylum abundances			
Treatment (Heated vs. Control)	1	304.34	0.3436
Soil types (Organic vs. Mineral)	1	953.16	0.0082^*^
Time points (T2 vs. T6)	1	117.66	0.8923
Treatment × Soil types	1	147.47	0.9807
Treatment × Time points	1	231.75	0.5343
Soil types × Time points	1	64.225	0.9807
Treatment × Soil types × Time points	1	310.91	0.3315
Residual	22	279.53	
Total	29		

* Indicates significant value of *p* at *α* = 0.05.

### Carbohydrate-Active Transcripts (CAZys)

**Figure 3:**
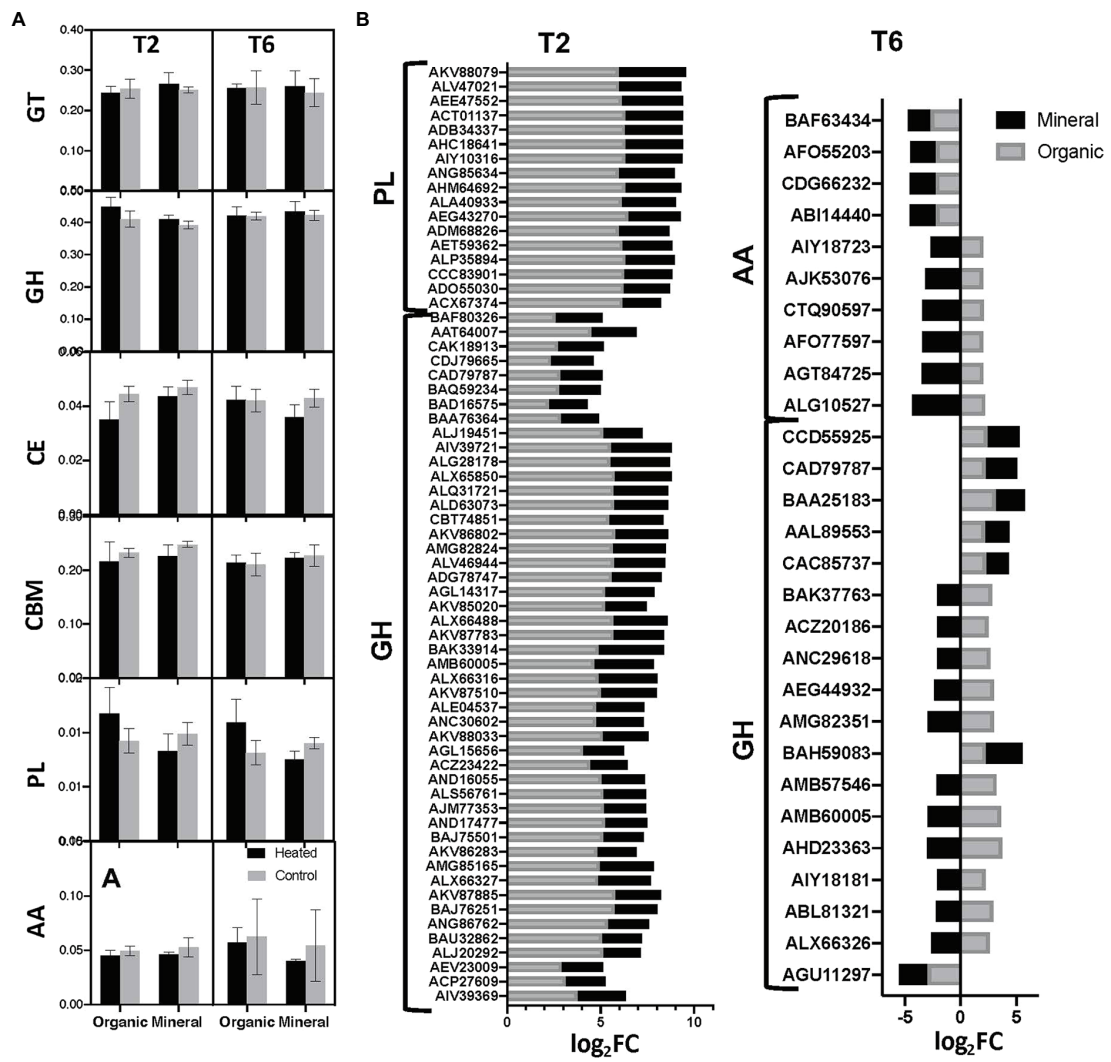
Carbohydrate active enzymes annotations – **(A)** mean relative abundances of transcripts annotated in the six enzyme classes according to the CAZy database. CAZy family codes: GT, glycosyltranferases; GH, glycoside hydrolases; CE, carbohydrate esterases; PL, polysaccharise lyases; CBM, carbohydrate binding modules; and AA, axillary activities (oxidative enzymes); **(B)** Stacked plot comparing the differential expression of individual CAZy annotated genes between heated and control plots within the two most abundant enzyme classes in organic and mineral horizons in T2 and T6 time points. Differential expression is measured as log of the FC. So, a log_2_FC of +2 means twice the abundance of the gene in heated plots compared to control, while a log_2_FC of −2 indicates twice the abundance of the gene in control plots compared to heated. All differential expression is measured at FDR 5%. Note that +ve and −ve FC ratios are relative to the specific heated and control plots that are used in the pair-wise comparisons.

To understand the dynamics of microbial C biotransformation in our sample, we annotated our transcripts using the CAZy database. About 5.24% of the filtered reads were attributed to CAZy classes (~85,000 reads per sample) and 316 distinct CAZy classes were identified. Similar to KEGG annotations, nMDS revealed no significant partitioning of samples based on treatment (H vs. C) or time-point (T2 vs. T6; Figure 1B). PERMANOVA analyses revealed a significant effect of horizon (M vs. O) with no interaction between treatment, horizon, and time-point (Table 3). Based on Cohen’s d effect size estimations, soil type had the largest effect on CAZy abundances followed by treatment and time-points (Table 2). Transcripts belonging to the six major enzyme classes showed no observable variation in abundances between heated vs. control plots (Figure 3A). However, *edge R* analyses revealed significant differences in their abundances between heated and control plots, most of which belonged to glycoside hydrolases (GH) enzyme class that hydrolyses glycosidic bonds in complex carbohydrates (Figure 3B). The top 150 most abundant CAZy families showed similar abundances across all samples (See Supplementary Data: Appendix 6). Further, similar to KEGG annotations, differentially expressed transcripts across samples were predominantly unique with only ~2.8% of annotated transcripts seen in more than one treatment conditions (See Supplementary Data: Appendix 7). Both these trends are perhaps not surprising, given that we did not observe significant clustering of transcripts in our ordination analyses (Table 3; Appendix 4).

In the spring time-point (T2), the two most abundant enzyme classes were GH and Polysaccharide Lyase (PL), with all transcripts showing a higher abundance in heated plots compared to control in both organic and mineral horizons (Figure 3B). In the autumn time-point (T6), the most abundant enzyme classes were GH and Auxiliary activities (AA) that includes mono-oxygenase and lignolytic enzymes (Figure 3B). In T6, the organic horizon showed an increased abundance of most GH and AA transcripts (80%) in heated plots, whereas in the mineral horizon the same transcripts were depleted in the heated compared to the control plots, resulting in 56.6% of the transcripts showing opposite patterns between the two soil layers (Figure 3B). Specifically, CAZymes belonging to GH families 3, 7, 11, 15, and 38, associated with complex carbohydrate metabolism targeting structural polysaccharides such as starch, cellulose, and glycogen and PL 11 associated with metabolism of cell wall derived polysaccharides showed higher abundances in heated plots in the organic soil. Mineral horizon had a less pronounced warming effect with most transcripts showing smaller fold changes (T6) and decreased abundances (T2) in the heated plots. Warming induced enrichment of GH 65 that is related to starch and dextrin metabolism was observed across all mineral soil samples.

### Taxonomic Survey Using Protein Coding and rRNA Genes

Based on rRNA taxonomic classification, we detected 35 phyla across 30 samples, with only eight appearing in every sample. On average, reads assigned to these eight phyla made up 80% of the total prokaryotes found in each sample. They were *Acidobacteria*, *Actinobacteria*, *Bacteroidetes*, *Crenarchaeota*, *Euryarchaeota*, *Firmicutes*, *Proteobacteria*, and *Verrucomicrobia*. The most highly represented phyla were *Acidobacteria*, averaging 17.8% of each community (See Supplementary Data: Appendix 8). Treatment effects (*p* < 0.05) were found for three phyla in organic horizon – *Actinobacteria* (*p*_0_ = 0.014, *F*_0_ = 7.9, *d*_0_ = 1.4) were enriched with chronic warming, while *Crenarchaeota* (*p*_0_ = 0.021, *F*_0_ = 6.7, *d*_0_ = −1.3), and *Planctomycetes* (*p*_0_ = 0.064, *F*_0_ = 1.5, *d*_0_ = −0.61) were depleted in heated compared to control soils (*p* is the probability of a given F-statistic arising from a null F-distribution, F is the F-statistic, and *d* is the Cohen’s d effect size of heat-treatment). All values reported are for treatment effect in organic soil; no phyla were found to have a significant treatment effect in mineral soil. Shannon’s diversity decreased significantly from 2.07 to 1.98 on average with heat-treatment only in the organic soil samples (*p* = 0.024). Similar to functional annotation, PERMANOVA analyses revealed a significant effect of horizon (M vs. O) with no significant interaction between treatment, horizon, and time-point (Table 1). Cohen’s d effect size estimations on phylum abundances showed highest effect of soil type (i.e., O vs. M) followed by treatment and time-points (Table 2). nMDS revealed no significant partitioning of samples based on treatment (H vs. C) or time-point (T2 vs. T6; Figure 1C).

**Figure 4:**
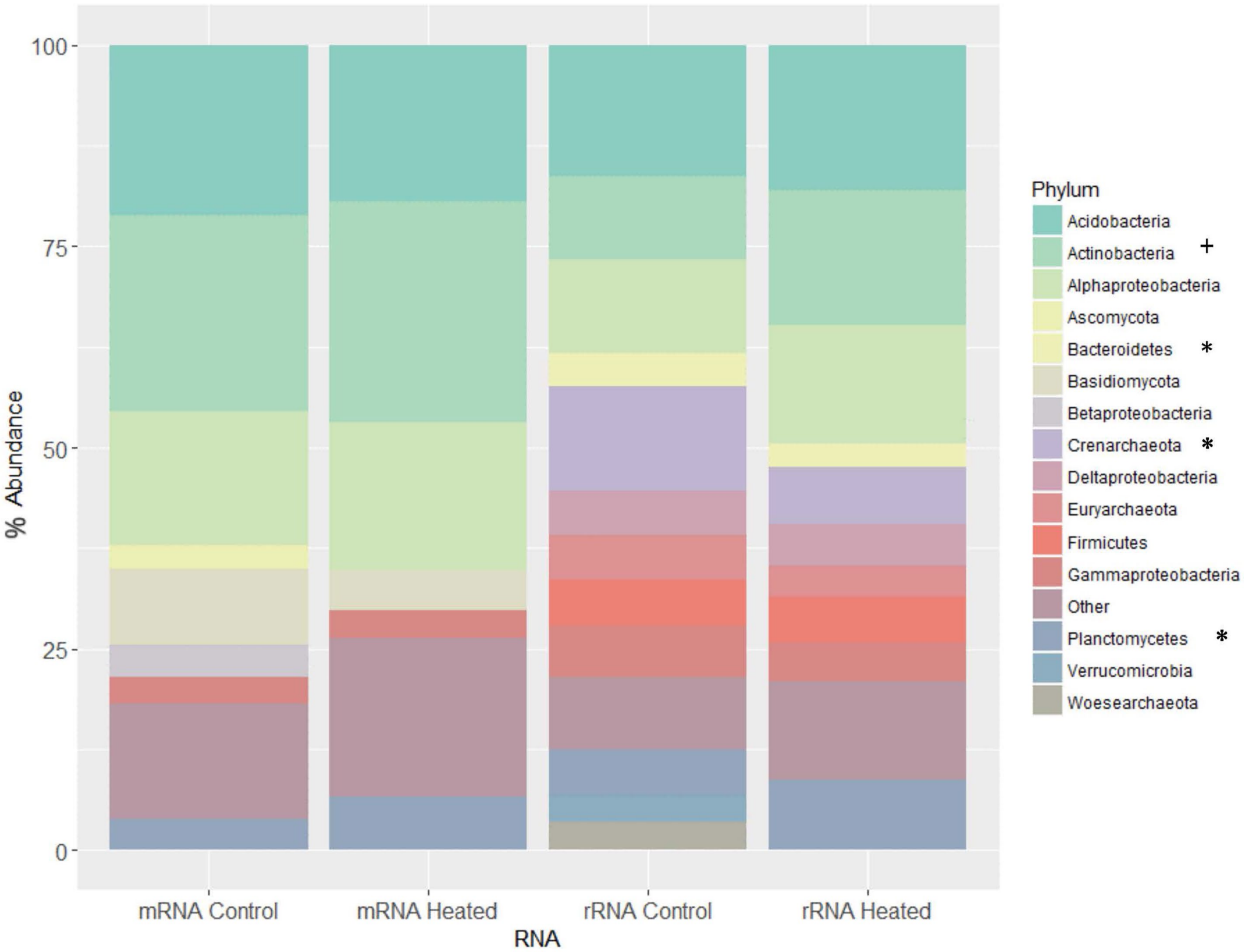
Taxonomic classifications – community composition of heated and control samples using taxonomic assignments from both rRNA and mRNA reads in Organic horizon. “+” indicates enrichment and “*” depletion of phylum in heated compared to control plots. rRNA and mRNA reads were annotated using MATAM and MEGAN programs, respectively. Composition is computed using relative abundance and all assignments are given at the phylum level besides those for proteobacteria, which are at the class level. Note that assignments to eukaryotic phyla occur only for mRNA (as rRNA assignments are performed based on alignments for the 16S sequence, which occurs only in prokaryotes). Only phyla/classes occurring at >3% on average are shown and are otherwise grouped into “Other.” Only for rRNA assignments did phyla in Archaea exceed the 3% threshold for being graphed; no Archaea cleared 0.1% abundance in the mRNA samples.

We annotated our putative mRNA in MEGAN using the LCA method to compare the differences in taxonomic assignments from rRNA and protein coding reads. mRNA taxonomic classification yielded similar results to rRNA community composition where the top three most abundant phyla were *Actinobacteria*, *Acidobacteria*, and *Proteobacteria* (Figure 4), although, the most highly represented phylum was *Actinobacteria*, averaging 18.9% of each community (See Supplementary Data: Appendices 8, 9). The relative abundances of these three dominant phyla did not show a significant difference across treatment, horizon, and time points. A total of 10 bacterial phyla appeared in all 30 samples that made up more than 81% of the total transcripts that were assigned a taxonomic classification. Treatment effects of *p* < 0.05 (compared to a null F-distribution) were found only in *Bacteroidetes* (*p*_0_ = 0.052, *F*_0_ = 4.529, *d*_0_ = 1.06) that showed depletion in heated plots compared to controls (Figure 4). Similar to rRNA annotation, no phyla showed a significant treatment effect in mineral soils. Shannon’s diversity was 1.99 in organic and 1.92 in mineral.

### Weighted Gene Correlation Network Analyses

**Figure 5:**
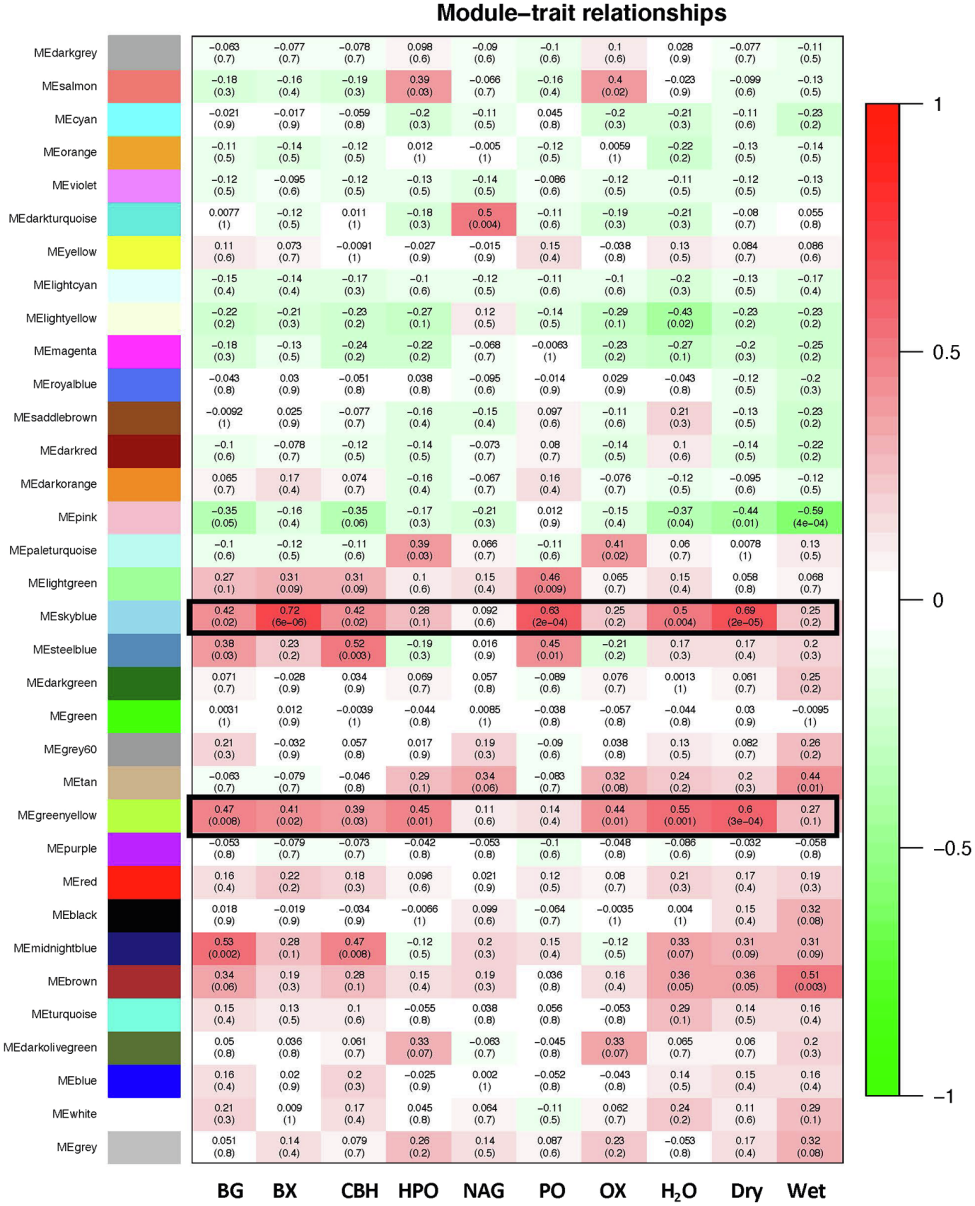
Co-weighted network analyses – module-trait associations between KEGG annotated transcript abundances and enzyme concentrations and physical soil parameters. Each row corresponds to a module eigen gene and each column represents a trait. The top number indicates the correlation (|cor|) between the eigen gene value and the individual trait and the bottom number indicates the *p*-value. The colors from green to red indicate positive or negative correlation, respectively. Enzyme codes – BG, β-glucosidase; BX, β-xylosidase; CBH, cellobiohydrolase; HPO, peroxidase; NAG, β-N-acetyl-glucosaminidase; PO, phenol oxidase; dry, dry weight of samples; and wet, wet weight of samples. Trait data are taken from Pold et al. (2017).

Weighted gene correlation network analyse (WGCNA) identified a total of 34 module eigen genes (ME), with skyblue (60 genes) and green yellow (169 genes) modules showing significant positive association with trait data (average module-trait *p* < 0.05; Figure 5). The co-expressed genes in these modules were found to be associated with important activities such as degradation of starch and complex carbohydrates, heat shock responses, and cellular respiration. Notably, enzymes related to the four hydrolytic (BG, BX, CBH, and NAG) and two oxidative enzymes (PO and HPO) measured in these soils included heat-resistant alpha-amylase (Vaseekaran et al., 2010); oxalate and hexediote decarboxylases; aminopeptidase, phosphorylase, and glutathione-independent formaldehyde dehydrogenase. However, transcripts annotated within any of these six enzyme classes included in our trait data (Pold et al., 2017) did not show any significant enrichment in either modules. Absence of such direct correlations indicates a complex link between gene expression and consequent phenotypic and physiological outcomes that necessitates additional screening of these processes in soil communities.

## Discussion

Forests are significant global C sinks (Pan et al., 2011; Zhu et al., 2019), and understanding their ecology helps to manage and predict C cycling processes and their associated feedback to global climate. Microbial decomposition of soil organic matter is expected to increase in response to warming. The rates and mechanisms of these microbial driven soil C losses are, however, not fully understood. In this study, we used transcriptome (functional) data to identify changes in the structure and function of soil microbial communities in response to 30 years of simulated warming. Previously, we observed increased total soil carbon loss (Melillo et al., 2017; Pold et al., 2017), and evidence of increased microbial activity and an increasing role of bacteria in soil C degradation (Pold et al., 2015, 2017). However, meta-transcriptomes revealed an overall small effect of chronic warming on bacterial gene transcript abundances (Figure 2; Table 3). Although, we did not see a significant warming effect in major KEGG and CAZy classes (Figures 2, 3), several transcripts of enzymes related to carbohydrate, fatty acid, and lipid metabolism exhibited higher abundances in heated plots, primarily in the organic horizon (Figures 2C, 3C). These results are consistent with previous studies that showed an increased concentration of lipids (Pold et al., 2017) and overall decline in the quality and quantity of SOM with 3 decades of warming in these forests (Bradford et al., 2008; Pold et al., 2015, 2017). Pold et al. (2016) reported “warming effect” on similar carbohydrate-degrading enzyme genes relative abundances as seen in this study, but in mineral horizons in soils collected in Phase III when respiration was slightly higher in the heated plots (Melillo et al., 2017). They suggested an incomplete degradation and subsequent translocation of SOM from organic into the mineral horizon. Earlier depletion of labile C in the organic layer might have led to functional adjustments in these communities that are now able to access more recalcitrant SOM. This can subsequently result in complete or near-complete degradation of organic matter within the top layer, accounting for the “warming effect” in organic horizon observed here on soils collected in Phase IV (respiration similar or equal between plots; Melillo et al., 2017). Previously, it has been observed that compared to mineral horizon, organic soil exhibits greater shifts in structural and functional potential in response to environmental pressures (Cardenas et al., 2015; DeAngelis et al., 2015). Further, changes in SOM quality and quantity impacts the functional diversity in soil microbes to a greater extent compared to taxonomic diversity (Baldrian et al., 2012; Uroz et al., 2013). Taken together, the observed functional shifts in organic layer communities toward increased carbohydrate and lipid degrading potential not only supports our initial hypotheses of increased degradation of organic matter in the heated plots, but also provides evidence of a more responsive organic soil type to long-term warming.

Microbial C-dynamics appear to be dominated by different metabolic processes during different seasons and soil layers (See Supplementary Data: Appendices 5, 7), as evidenced by the fact that across both time points and horizons, the shifts in these transcript abundances are mostly unique. These differences in microbial processes can be due to variations in soil microclimatic parameters leading to transient changes in resource availability driven by diurnal, seasonal, and annual changes in these forests (Luo and Zhou, 2006; Edwards et al., 2007; Blankinship et al., 2011; Contosta et al., 2011; Zhang et al., 2011). Peng et al. (2018) found that changes in microclimatic parameters were the primary drivers of loss in soil organic carbon in a grassland ecosystem in the Qinghai-Tibet Plateau. Additionally, WGCNA analyses indicated a complex relationship between gene abundances and community function, where although several transcripts showed strong association to soil enzyme activities, no direct correlation was observed (Figure 5). This decoupling can be the result of microclimatic and temporal variability between samples, although, the broad substrate specificity of the KEGG enzymes cannot be ignored as a possible explanation for the same. Therefore, additional annual and seasonal time points should be sampled to further test whether the same processes dominate the microbial C-cycling consistently over time. Nevertheless, such spatial and temporal heterogeneity in microbial C-cycling genes can affect the functional capacities of soil overtime and is crucial to understand and predict warming-induced C losses in a warming climate.

While changes in gene expression based on chronic warming were not large, long-term warming caused a substantial decrease in microbial biomass (Frey et al., 2008; DeAngelis et al., 2015) and changes in abundance of certain microbial populations (DeAngelis et al., 2015; Pold et al., 2016). In this study, we used both mRNA and rRNA to identify changes in bacterial communities in response to warming (Figure 4). Our results showed that mRNA and rRNA annotations provided similar estimates of the dominant phyla, except that *Actinobacteria* were overrepresented in the mRNA sequences, possibly suggesting their increased metabolic activity relative to other groups. mRNA annotations were not able to classify the broad diversity that the rRNA was able to capture especially for rarer phyla such as *Crenarcheota*, *Firmicutus*, and *Deltaproteobacteria*. Cohen’s d values for mRNA and rRNA varied between soil types, which might be due to differences in annotation techniques used in either case (See Supplementary Data: Appendices 7, 8). Our data therefore suggests that compared to rRNA classification, taxonomic annotation of protein coding reads can only provide a cursory estimate of the abundant soil bacterial communities.

By separately analyzing rRNA vs. mRNA, we were able to detect changes in function that are separate from changes in community structure. Consistent with previous studies (DeAngelis et al., 2015; Pold et al., 2016), we observed relatively small changes in community structure with warming (Figure 1C), though both methods are biased toward over-representing bacteria. *Actinobacteria* increased, while the phyla *Crenarchaeota*, *Planctomycetes*, and *Bacteroidetes* decreased in relative abundance in the heated plots compared to controls (Figure 4). Changes in phyla abundance were only observed in the organic horizon, with a significant decrease in diversity in the heated plots. The three phyla depleted with long-term warming were all Gram-negative, and the enriched *Actinobacteria* were Gram-positive, supporting the previous observation of community shifts toward Gram-positive in the heated plots (Frey et al., 2008). The increased abundances of *Actinobacteria* might indicate a greater role of this taxa in driving the carbohydrate and lipid degradation in the heated plots. Although, we did not observe any differences in ligninolytic enzyme activities in our functional data, several members of *Actinobacteria* are associated with production of bacterial laccases that are important enzymes in lignin degradation (de Gonzalo et al., 2016). As such, their increase in the heated plots might imply a shift toward communities that are able to access more recalcitrant SOM, such as lignin and lignin-derived SOM, as labile C pools continue to deplete in response to warming. Members of *Actinobacteria* are generally over-represented in genomic databases; but, the consistent enrichment of *Actinobacteria* in heated plots in the present and other warming studies (Castro et al., 2010; Sheik et al., 2011; Shade et al., 2012) might indicate a greater tolerance to warming and its associated changes in soil physical parameters (Barnard et al., 2013).

Long-term warming in these forests has resulted in a decrease of fungal biomass with no substantial change in bacterial biomass in response to warming (Frey et al., 2008; DeAngelis et al., 2015). Enzyme activities were observed to be the same per gram of soil but decreased per unit biomass because of warming-induced decline in microbial biomass (Pold et al., 2017). Further, long-term warming also decreases soil moisture by lowering the soil water holding capacity (Rustad et al., 2001; Werner et al., 2020), which in turn can drive changes in microbial biomass and enzyme productions. While gene expression on a per gram soil basis may show small changes, as we present here, these changes are probably occurring against a backdrop of a smaller or less abundant microbial community in the heated plots. The use of internal standards was an attempt to distinguish between increased functional capacity vs. functional shifts in microbial communities by measuring absolute transcript copy numbers (Conant et al., 2011). However, we were unable to estimate the absolute transcript copy numbers as four of the 32 samples showed no recovery of internal standards (Table 1). This could be due to differences in sequencing artifacts, which can be avoided in the future by either increasing technical replicates or using a combination of multiple internal standards to increase robustness (van de Peppel et al., 2003). Nonetheless, measuring absolute transcript copy number in future functional studies will be valuable to estimate not only the role of under-represented but crucial metabolic pathways (Gifford et al., 2011), but also to evaluate the role of resilience and/or adaptation in functional potential of these communities in response to chronic long-term warming. To our knowledge, this is the first report on the application of internal standards to measure absolute transcript abundances in soil systems. Further refinement would be needed to determine if this method is applicable to soil systems.

Seasonal variation in abiotic factors, such as temperature and moisture content, can both directly and indirectly impact soil C dynamics (Benbi et al., 2014; Lladó et al., 2017; Žifčáková et al., 2017) and changes in soil microbial community (Habekost et al., 2008; Cao et al., 2011). We therefore predicted a seasonal impact on warming-accelerated microbial decomposition. We chose two time points, T2 (June) and T6 (October), based on Pold et al. (2017) that demonstrated a strong seasonal trend in extracellular enzyme activities, with most enzymes peaking during T2 and showing a decline or a 2nd peak during T6. Contrary to our expectations, we detected little effect of season on community response and community structure. In fact, the seasonal effect was the smallest factor driving global gene expression, carbohydrate-associated gene expression, and taxonomic marker transcripts (Table 2). Our results are consistent with another meta-transcriptome study from the same site that found no seasonal variation in community structure across six different time points (Rodríguez-Reillo, 2019). Previous studies have reported increased respiration and enzyme activities in summer (highest temperature), with lower microbial decomposition rates in spring and winter (lowest temperature; Contosta et al., 2011; Žifčáková et al., 2017). Winter communities decomposed more recalcitrant substrates such as lignin and cellulose, as compared to summer communities (Koranda et al., 2013; Žifčáková et al., 2017). To our knowledge, studies on seasonal variations on microbial gene expression (meta-transcriptome) are still lacking. The absence of a seasonal effect in this study is possibly due to the collection of soils at time points that were experiencing similar temperatures at the time of sampling (Pold et al., 2017). Further, seasonal variation in microbial activity is related to availability of different C-sources (Contosta et al., 2011), which can decrease under long-term warming due to depletion of labile C and reduction in microbial biomass (Frey et al., 2008). This, however, cannot explain the lack of seasonal variation in control plots. Furthermore, Žifčáková et al. (2017) indicated that there is temporal variation in bacterial vs. fungal gene expression related to decomposition of recalcitrant C-pools. Comparing the bacterial gene expression from this study with fungal gene expression (Romero-Olivares et al., 2019) across these time points might reveal seasonal patterns in microbial functions that are otherwise obscured. Additionally, extrapolating seasonal trends from gene expression measured at a single time point is perhaps not feasible given the observed diurnal variations in abiotic and biotic factors in temperate ecosystems (Zhang et al., 2011). In the future, gene expression should be measured and compared recurrently over extended periods to understand and capture changes in microbial functional potential in response to long-term chronic warming. Although, analyses of such vast sequencing data are technologically challenging, understanding, and capturing the nuances in microbial processes will help us better predict microbial driven C cycle-temperature feedbacks in the future.

## Conclusion

In this study, we did a comparative meta-transcriptome analysis to understand the consequences of long-term warming on terrestrial soil bacterial communities and the related effects on soil C dynamics. Although, the warming effect on overall gene expressions was small, we did see increased expression of several enzymes involved in carbohydrate and lipid metabolism in heated plots, providing support to our main hypothesis of warming-induced acceleration of recalcitrant C-pool. Our results also show a strong effect of soil type on carbohydrate-associated gene expression, with greater warming effect on microbial transcription in the top organic horizon. We did not find any substantial changes in microbial community structure in response to warming. Both community structure and function showed no seasonal variation in our data. The meta-transcriptome approach allowed us to identify unique metabolic processes that controlled microbial decomposition and in turn soil C fluxes in different soil types in response to long-term warming. The lack of an overall seasonal gene expression pattern in this study highlights the need for comparing microbial functional profiles over an extended period of time to understand the contribution of these mechanisms to soil C-cycling in a chronically warmed forest. Information as such will substantially increase our predictive power to model correlated changes in microbial functions and temperature fluctuations in a warming climate.

## Data Availability Statement

The Next-seq RNA sequence data from this study have been uploaded to the DDBJ Sequence Read Archive (SRA) under the accession number PRJNA624774.

## Author Contributions

KD and JB conceived and designed the study. JM provided access to the study site and assisted with soil sampling. AB conducted RNA isolation from samples. PR and LA conducted library construction. PR performed Illumina sequencing and data analyses. SG performed the taxonomic analyses. JS performed part of the CAZy analyses. PR and KD wrote the paper. All authors contributed to the article and approved the submitted version.

## Conflict of Interest

The authors declare that the research was conducted in the absence of any commercial or financial relationships that could be construed as a potential conflict of interest.

## Publisher’s Note

All claims expressed in this article are solely those of the authors and do not necessarily represent those of their affiliated organizations, or those of the publisher, the editors and the reviewers. Any product that may be evaluated in this article, or claim that may be made by its manufacturer, is not guaranteed or endorsed by the publisher.
