# Supplementary material for: The Transcriptional Response of Soil Bacteria to Long-Term Warming and Short-Term Seasonal Fluctuations in a Terrestrial Forest
Source: Front Microbiol. 2021 Aug 27;12:666558. doi: 10.3389/fmicb.2021.666558 (PMC8429792; doi:10.3389/fmicb.2021.666558)

Supplementary Data: Appendix 1: Additional description of protocols for synthesis, purification and identification of the internal RNA standard.

Synthesis and purification of the Internal RNA Standard. Briefly, the standard was constructed by linearizing pET-41 plasmid with BamHI restriction enzyme (New England Biolabs, Ipswich, MA). The engineered RNA standard would allow us to distinguish it from any biological sequences endemic to the soil. Complete digestion of plasmid was verified with 1% Agarose gel electrophoresis following cleaning with a phenol-chloroform-isoamyl alcohol purification system. The sticky 5' overhangs were removed using Mung Bean Nuclease following manufacturer's instructions (Promega, Madison, WI). The cleaned fragment was then transcribed using the Riboprobe *in vitro* Transcription Kit (Promega, Madison, WI) according to the manufacturer's instructions using a T7 RNA polymerase to create a 943 nt long RNA fragment. The purified RNA standard was then quantified using Qubit RNA BR assay (Qubit 2.0 Fluorometer, Invitrogen, CA) and then diluted using DEPC-treated water before adding to samples.

RNA Internal Standard Identification. Internal standards were quantified and filtered from the raw reads using BBDuk with default parameters that compared reads to the kmers in a reference sequence (BBTools, Joint Genome Institute, CA; http://jgi.doe.gov/data-and-tools/bbtools/). Following quantification of the number of internal RNA standards in each sample, the total transcript pool and individual transcript abundances were calculated using the equations $P_a = P_s X S_a / S_s$, and $T_a = T_s X P_a / P_s$, where, P_a = total transcripts in the sample, P_s = protein encoding reads in the sample, S_a = molecules of internal standard added to the sample, S_s = number of internal standard reads identified, T_a = total molecules of any particular transcript in the sample, and T_s = number of transcripts of interest in the sample (Satinsky et al., 2013).

Supplementary Data: Appendix 2: Three-way ANOVA showing no significant effect of treatment (heated and control); horizon (organic and mineral); time points (T2 and T6) and their interaction thereof on **(A)** putative mRNA yield and **(B)** internal standard sequence reads **(C)** annotated KEGG and **(D)** annotated CAZy transcripts for all the 31 metatranscriptome libraries used in this study.

(A) ANOVA Table for total putative mRNA

Source of Variation	% of total variation	P value	P value summary	Significant?
Treatments	8.312	0.0879	ns	No
Time Points	9.459	0.0703	ns	No
Soil types	8.661	0.082	ns	No
Treatments x Time Points	9.429	0.0707	ns	No
Treatments x Soil Types	7.616	0.101	ns	No
Time Points x Soil Types	7.592	0.1015	ns	No
Treatments x Time Points x Soil Types	8.687	0.0816	ns	No

(B) ANOVA Table for total Internal Standard Sequences

Source of Variation	% of total variation	P value	P value summary	Significant?
Treatment	4.3	0.2425	ns	No
Time points	1.362	0.5043	ns	No
Soil types	3.59	0.0678	ns	No
Treatment x Time points	10.97	0.0704	ns	No
Treatment x Soil types	0.5503	0.67	ns	No
Time points x Soil types	0.001578	0.9817	ns	No
Treatment x Time points x Soil types	0.1982	0.7978	ns	No

(C) ANOVA Table for KEGG annotation

Source of Variation	% of total variation	P value	P value summary	Significant?
Treatments	5.129	0.2315	ns	No
Time Points	0.1075	0.8603	ns	No
Soil types	3.602	0.3138	ns	No
Treatments x Time Points	10.25	0.0957	ns	No
Treatments x Soil Types	1.220	0.5547	ns	No
Time Points x Soil Types	2.181	0.4312	ns	No
Treatments x Time Points x Soil Types	0.1263	0.8487	ns	No

(D) ANOVA Table CAZy annotation

Source of Variation	% of total variation	P value	P value summary	Significant?
Treatment	1.042	0.5796	ns	No
Time points	0.2028	0.8064	ns	No
Soil types	10.93	0.0818	ns	No
Treatment x Time points	1.840	0.4628	ns	No
Treatment x Soil types	9.319	0.1064	ns	No
Time points x Soil types	0.3101	0.7620	ns	No
Treatment x Time points x Soil types	0.3292	0.7550	ns	No

Supplementary Data: Appendix 3: Metabolic Pathways identified in iPATH (Letunic et al., 2008) that were represented by sequences annotated in the KEGG database in all 31 samples combined to ensure sufficient dept of sequencing and annotation coverage.

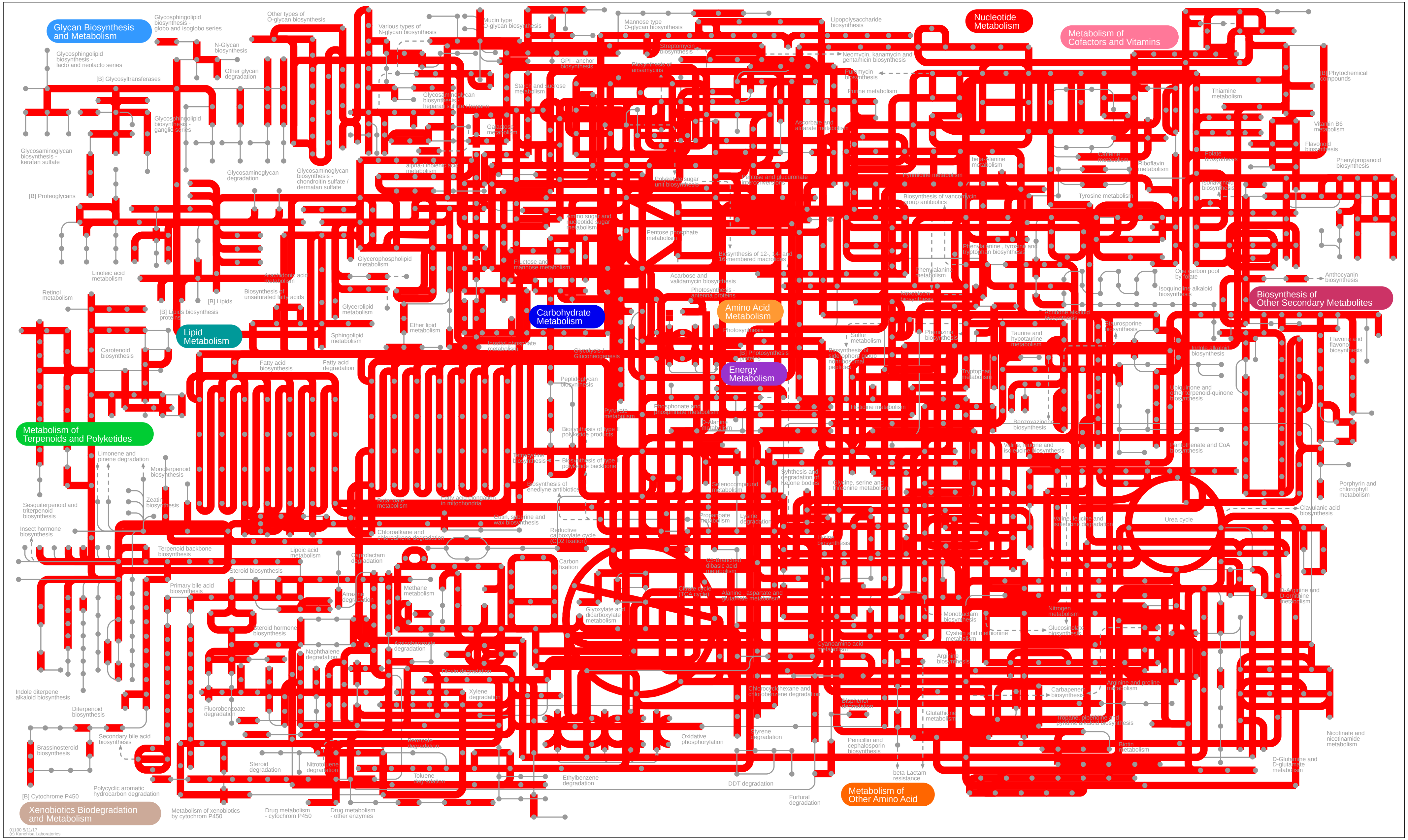

Supplementary Data: Appendix 4: Metabolic Pathways identified in iPATH (Letunic et al., 2008) that were differentially regulated in all samples between control and heated plots in T2-Organic **(A)** T2-Mineral **(B)** T2-Organic **(C)** T6-Mineral and **(D)** T6-Organic. Pathways in red shows a "warming effect" as these are upregulated in heated plots, pathways in blue show different direction of gene expression between heated and control paths. Differential expression is expressed in fold change ratios calculated in *Edge R*.

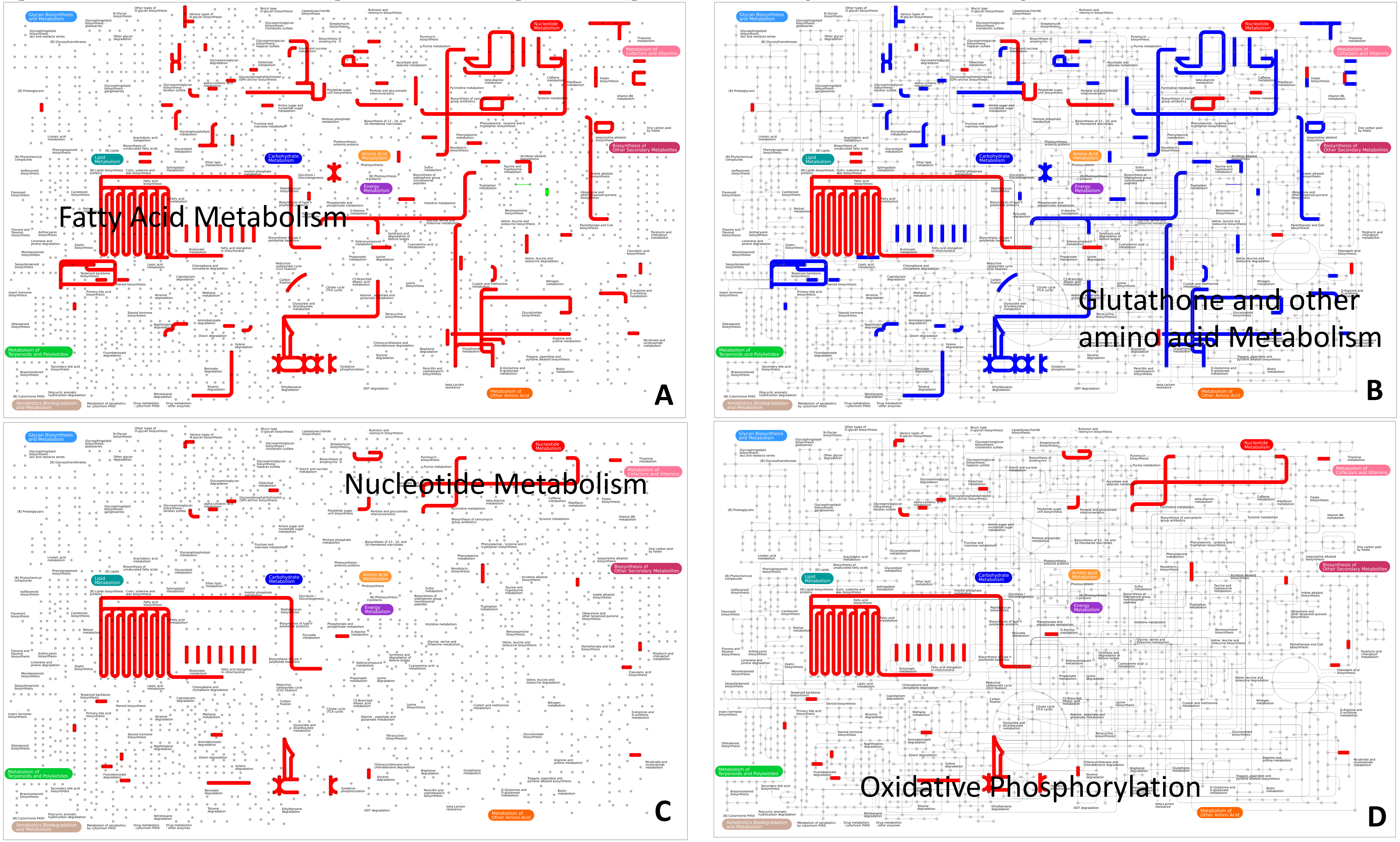

Supplementary Data: Appendix 5: Venn Diagrams showing the number of differentially expressed KEGG annotated genes that are unique as well as shared across different samples. **(A)** Shows the number of differential expressed genes between control and heated in (1) T2 – Mineral (Violet circle), (2) T2 – Organic (Yellow circle), (3) T6 – Mineral (Green circle), (4) T6 – Organic (Pink Circle); **(B)** Shows the number of differential expressed genes between time points T2 and T6 in (1) Mineral – Control (Violet circle), (2) Mineral – Heated (Yellow circle), (3)Organic – Control (Green circle), (4) Organic – Heated (Pink Circle); **(C)** Shows the number of differential expressed genes between Organic and Mineraal in (1) T2 – Heated (Violet circle), (2) T2 – Control (Yellow circle), (3) T6 – Control (Green circle), (4) T6 – Heated (Pink Circle).

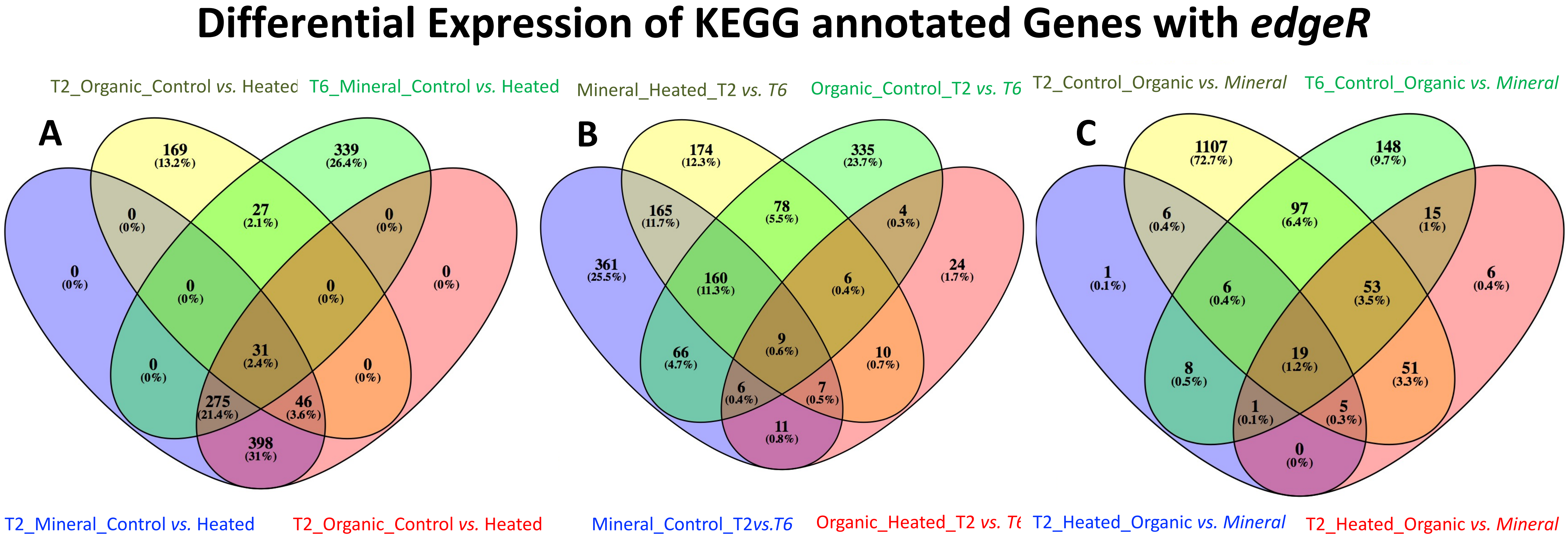

Supplementary Data: Appendix 6: Heat map of the 150 most abundant CAZy transcript abundance. The 150 most abundant CAZy families are presented in the heatmap to show variations in abundances across samples. CAZy family codes: GT, glycosyltranferases; GH, glycoside hydrolases; CE, carbohydrate esterases; PL, polysaccharise lyases; CBM, carbohydrate binding modules; AA, axillary activities (oxidative enzymes). The dendrogram clusters the CAZy by common expression patterns using a two-way hierarchical clustering using the complete linkage method. Transcripts annotated in each sample was normalized to the number of reads per million annotated mRNA in that sample.

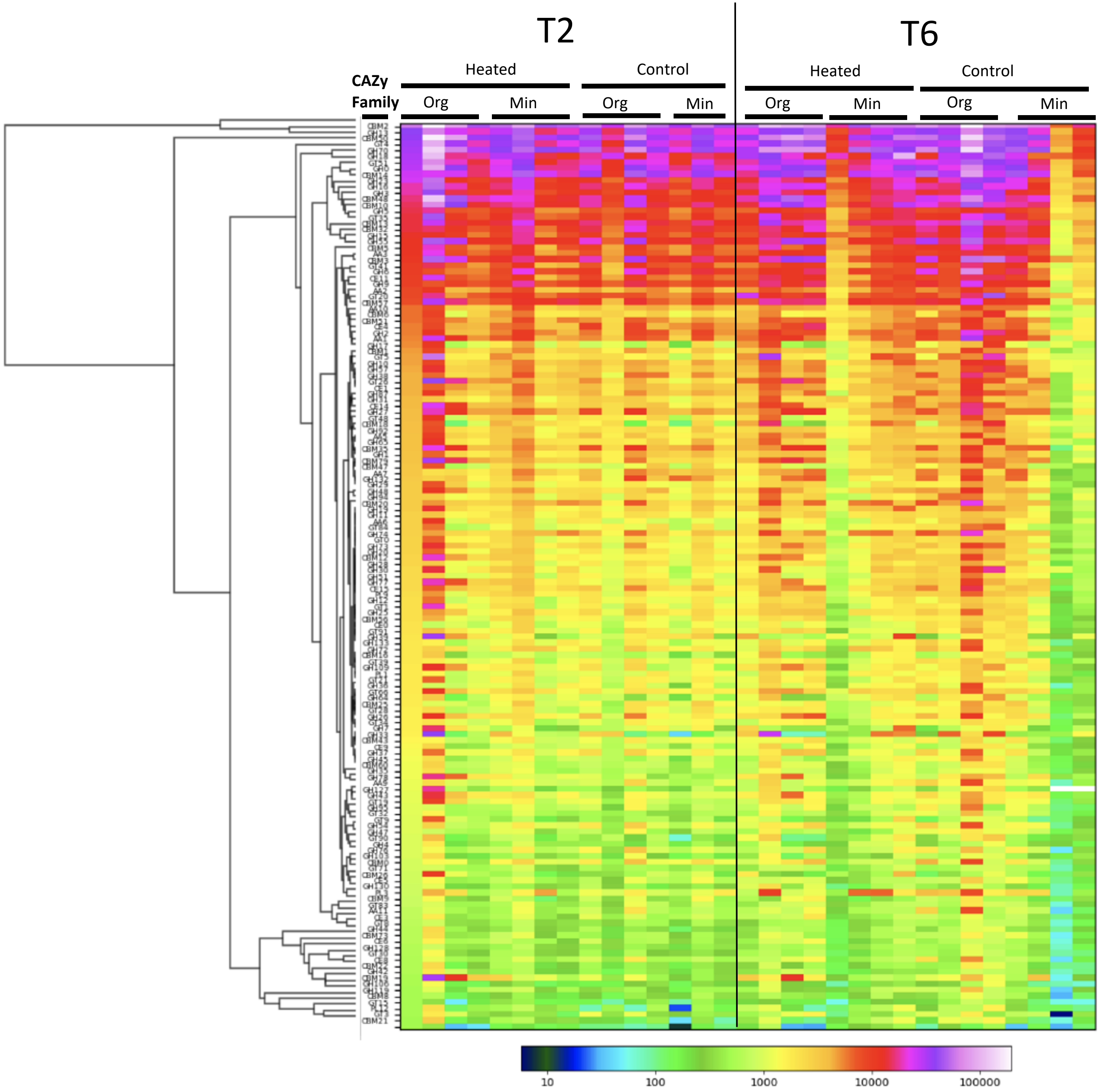

Supplementary Data: Appendix 7: Venn Diagrams showing the number of differentially expressed CAZy annotated genes that are unique as well as shared across different samples. **(A)** Shows the number of differential expressed genes between control and heated in (1) T2 – Mineral (Yellow circle), (2) T2 – Organic (Green circle), (3) T6 – Mineral (Blue circle), (4) T6 – Organic (Pink Circle); **(B)** Shows the number of differential expressed genes between heated and control that is common as well as unique between the organic and mineral soil horizons across the two time points T2 and T6.

Differential Expression of CAZy with *EdgeR*

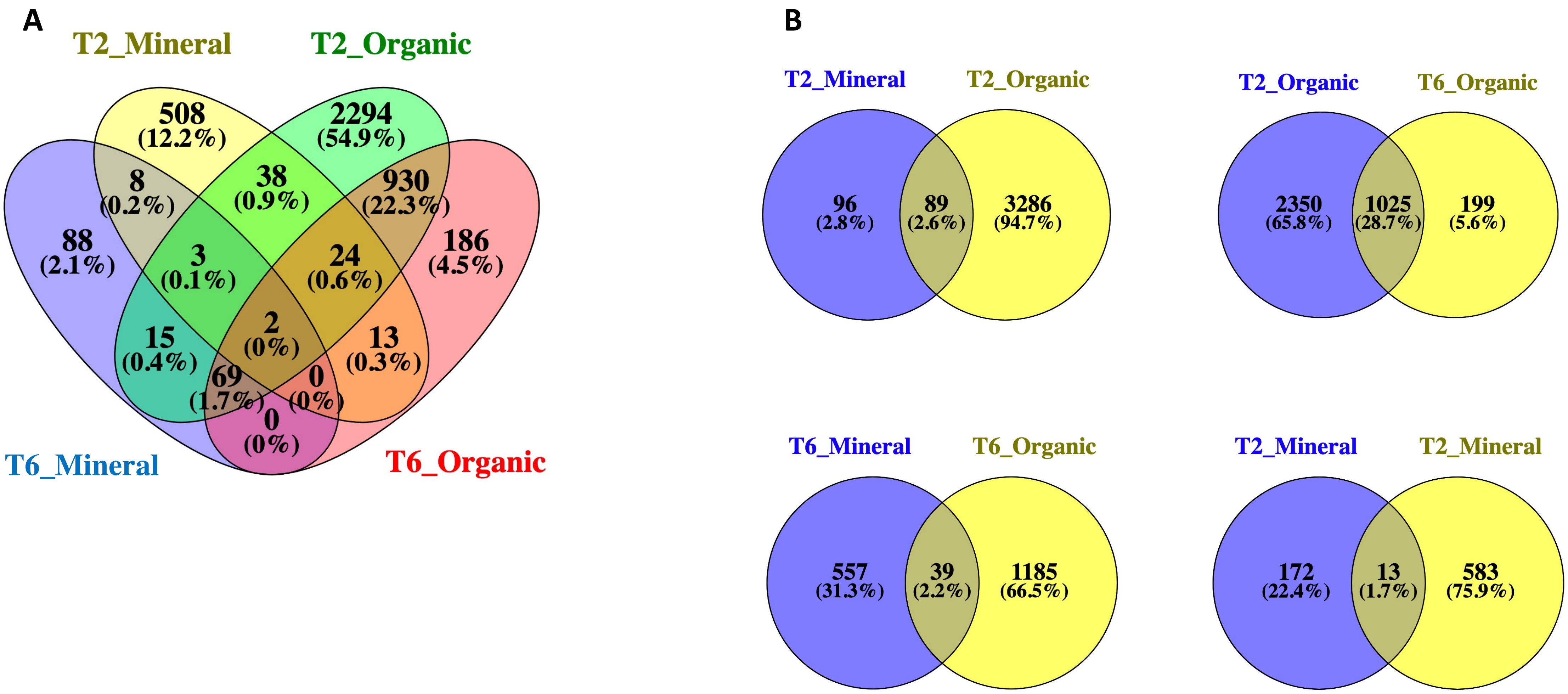

Supplementary Data: Appendix 8: Top row compares percent abundances between phyla for each soil type. Percent abundance for a phylum is computed as the percentage of short reads from a given sample assigned to that phylum by MATAM. Only the 8 phyla whose average % abundance exceeds 3 are explicitly plotted, while the remaining 27 are grouped into "Other". Inside lines on boxes are medians, edges are 25th and 75th percentiles, and whiskers extend to 1.5 times the interquartile range. Outliers are discarded. Asterisks ('*') above box plots for a phylum indicate that the % abundance probability density for that phylum is plotted. The second and third rows show average % abundances sampled from the posterior distribution described in Eq. 5. Each density curve is the result of about 100,000 simulations. There were 7 observations for each phylum in each treatment group from mineral soil samples, and 8 observations for each phylum in each treatment group

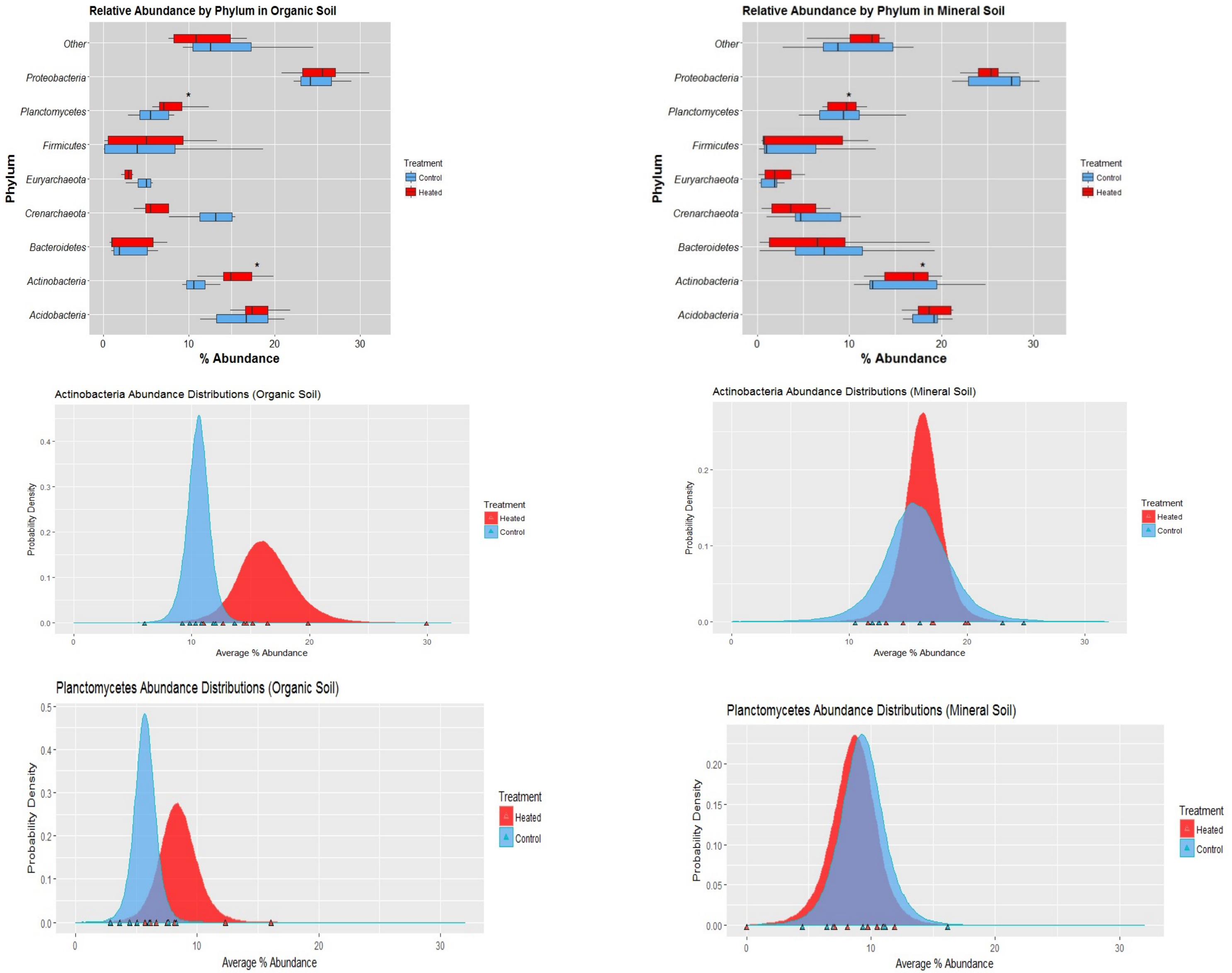

Supplementary Data: Appendix 9: (Upper Panel) Relative Abundances of the top 3 dominant bacterial taxa – Actinobacteria, Proteobacteria and Acidobacteria based on protein coding reads (putative mRNA) annotated by MEGAN v6 (Huson et al., 2016) using the LCA approach for taxonomic assignment using top 10% of hits and a minimum bit score of 50 as search criteria. (Lower Panel). Each bar represents the differences in abundances between the heated and control in organic and mineral horizons. Violet bars represent T2 and Blue bars represents T6 time-points respectively. +ve values means higher abundances in heated plots and –ve means higher abundances in control plots.

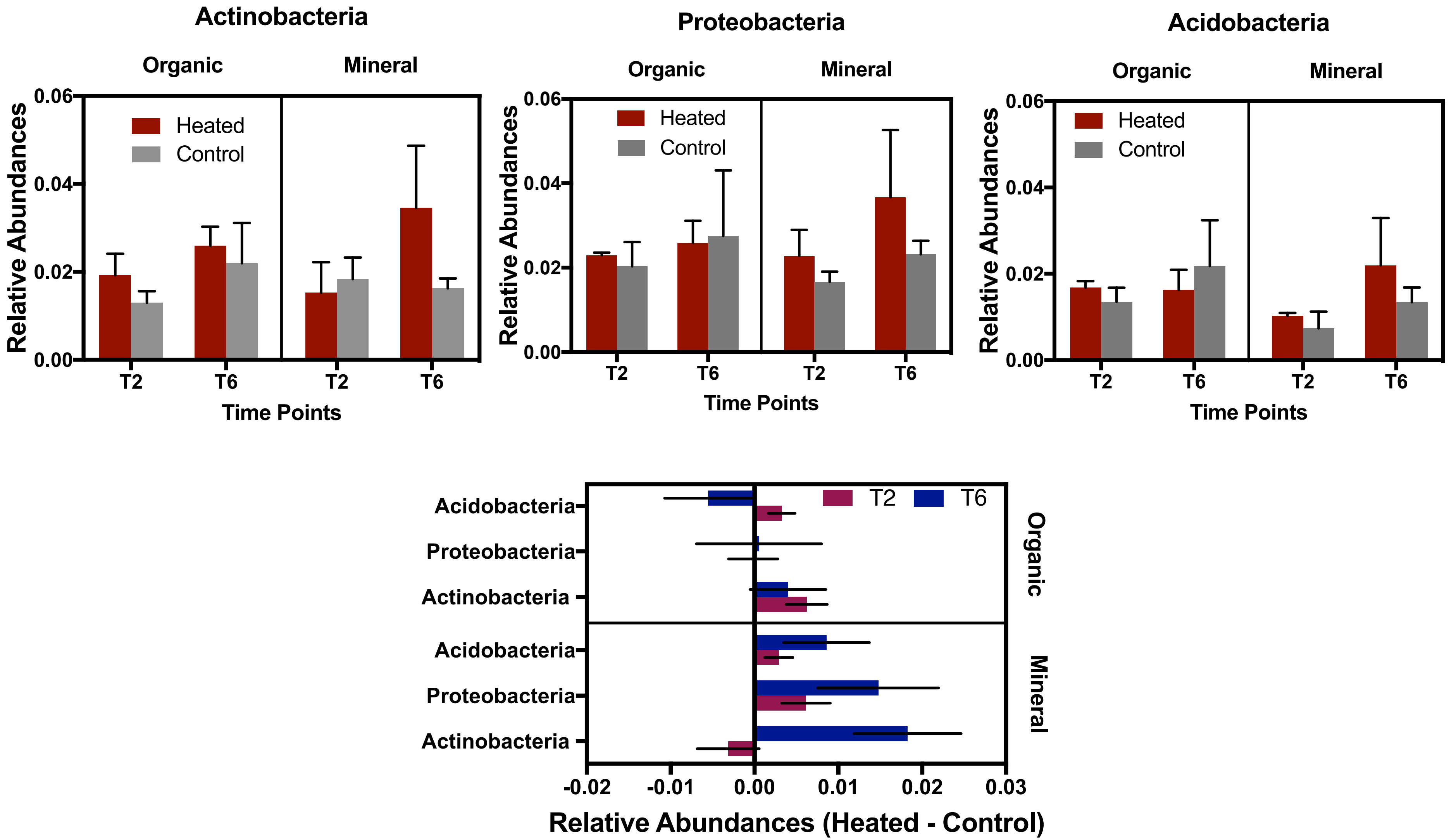

Supplement: Supplementary file 1 [file Data_Sheet_1.PDF]
